# Serum nerve growth factor in horses with osteoarthritis‐associated lameness

**DOI:** 10.1111/jvim.16718

**Published:** 2023-04-21

**Authors:** Anna Kendall, Claudia Lützelschwab, Johan Lundblad, Eva Skiöldebrand

**Affiliations:** ^1^ Division of Pathology, Pharmacology and Toxicology, Department of Biomedical Sciences and Veterinary Public Health Swedish University of Agricultural Sciences Uppsala Sweden; ^2^ Department of Anatomy, Physiology and Biochemistry Swedish University of Agricultural Sciences Uppsala Sweden

**Keywords:** equine, NGF, osteoarthritis, pain

## Abstract

**Background:**

Nerve growth factor (NGF) is a neurotrophin that is increased in osteoarthritic joints of horses. In humans, NGF has been associated with pain, and both synovial and serum NGF concentrations are increased in osteoarthritic patients. Studies in humans also have shown that serum NGF concentration can increase with stress. Serum NGF concentration should be evaluated in horses with osteoarthritis‐associated lameness.

**Objectives:**

Quantify and compare serum NGF concentration in horses with osteoarthritis‐associated lameness and sound horses. Additionally, the impact of short‐term stress on serum NGF concentration was investigated.

**Animals:**

Lame horses with radiographic evidence of osteoarthritis (n = 20), lame horses without radiographic changes in the affected joint (n = 20) and sound horses (n = 20). In addition, horses with acute fractures (n = 9) were sampled. To determine the effect of stress, serum from horses subjected to a stressful event (transportation, n = 5; stress confirmed by increased serum cortisol concentration) was analyzed.

**Methods:**

Cross‐sectional clinical study (lame, sound, and fracture cohorts) and experimental longitudinal study (stress cohort). Serum NGF concentration was determined using a quantitative sandwich ELISA.

**Results:**

Serum NGF concentration was increased in lame horses with radiographic evidence of osteoarthritis (*P* < .0001; median, 238 pg/mL; interquartile range [IQR], 63‐945 pg/mL) and in lame horses without radiographic evidence of osteoarthritis in the painful joint (*P* < .05; median, 31 pg/mL; IQR, 31‐95 pg/mL) compared with sound horses (median, 31 pg/mL; IQR, 31‐46 pg/mL). Serum NGF concentration did not increase with short‐term stress and was low in horses with fracture‐associated pain.

**Conclusions and Clinical Importance:**

Serum NGF concentration was high in the cohort with advanced osteoarthritis and should be investigated as a marker for osteoarthritis‐associated pain.

**Abbreviations**
HPA‐axis: hypothalamic‐pituitary‐adrenal axis
mNGF: mature nerve growth factor
NGF: nerve growth factor
p75^NTR^
: pan‐neurotrophin receptor
proNGF: proforms of nerve growth factor
ROC: receiver operating characteristic
TrkA: tyrosine kinase A

## 1 INTRODUCTION

Measuring pain in the horse is difficult. The inability to recognize subtle signs of pain can lead to situations in which horses are in painful states that go unnoticed by owners and veterinarians. Research in pain evaluation in horses has focused mainly on acute pain,^1^ but more centralized, chronic pain states are equally important. In horses, behaviors caused by osteoarthritic pain may be mistaken for frowardness,^2^ delaying appropriate veterinary investigation and intervention. In humans, the chronic pain state associated with osteoarthritis causes a substantial decrease in quality of life, and chronic pain can be considered a disease in itself (https://www.iprcc.nih.gov/sites/default/files/documents/NationalPainStrategy_508C.pdf, downloaded on 7/9/2022). Currently, no validated methods are available to evaluate low‐grade, centralized, chronic pain in horses, and more research into the subject is needed.

Nerve growth factor (NGF) is a neurotrophin that was first discovered in the 1950s.^3^ During embryonic development, neurons are dependent on NGF for survival and growth.^4^ Postnatally, the effects of NGF change. In addition to being associated with inflammation via stimulatory effects on cells of the immune system,^5^ NGF is a signaling molecule for pain.^6^, ^7^ In humans, NGF is increased in serum of patients with osteoarthritis compared with healthy controls.^8^, ^9^ Osteoarthritis‐associated pain is a combination of local processes in the joint (nociceptive pain) and a chronic, centralized (neurogenic) pain state.^10^ Because NGF concentration has been determined to increase in both of these processes, it likely plays a central role in osteoarthritis‐associated pain. Monoclonal anti‐NGF antibody treatment has been shown to attenuate osteoarthritis‐associated pain in humans, dogs, and cats.^11^, ^12^, ^13^ Because the inhibition of NGF can be used to treat osteoarthritis‐associated chronic pain, measurement of NGF may be useful in detecting such pain. However, serum NGF concentration also has been reported to increase with emotional stress in humans and after intraspecific fighting episodes in rodents.^14^, ^15^ To evaluate if serum NGF concentration shows promise as a marker for osteoarthritis‐associated pain in horses, stress‐related changes must be ruled out as a confounder.

Our aim was to quantify serum NGF concentrations in horses with lameness associated with advanced osteoarthritis and compare these to serum concentrations in horses with milder disease and in young healthy (sound) horses. Our aim also was to measure NGF concentrations in horses with acute pain and to determine if short‐term physiological stress influences serum NGF concentrations in horses.

## 2 MATERIALS AND METHODS

### 2.1 Samples

Advanced osteoarthritis group (n = 20): Horses presented for evaluation of lameness with a lameness grade or a reaction to flexion tests of ≥1 on a scale of 5^16^ were included if they had radiographic changes consistent with osteoarthritis (osteophytes) in ≥1 joints of the lame limb.

Lame, radiographically normal group (n = 20): Horses presented for evaluation of lameness or poor performance were included if they had a reaction to flexion test of ≥1 on a scale of 5^16^ and gait asymmetry detected on objective motion analysis (Equinosis Lameness Locator, Equinosis, Columbia, Missouri). The reaction to flexion and gait asymmetry were decreased in the lame limb after intra‐articular anesthesia with carbocaine. For the horse to be included, no radiographic changes could be present in the affected joint.

Healthy group (n = 20): Young horses with no signs of lameness were sampled before commencing training. The horses were examined by a veterinarian and no lameness was detected on dynamic evaluation and flexion tests before inclusion.

The flexion tests in all groups were performed as whole‐limb flexion as well as a fractionated distal limb flexion for 60 seconds. Horses were excluded from the study if they had received any treatment with corticosteroids in the 3 months before sampling.

Fracture group: Horses presented for acute, non‐weight bearing lameness or with a history of a traumatic event with clinical signs of pain (eg, pain face, reluctant to move, resenting palpation of the injured area) were included if they had radiographic evidence of acute fractures.

Sample size was based on a previous study^8^ and capped for convenience as clinical patients were studied. Serum samples were obtained by venipuncture of the jugular vein. Blood was allowed to clot and serum separated after centrifugation. Serum samples either were frozen immediately at −80°C or kept for a few days at −20°C until moved to −80°C for storage until analysis.

### 2.2 Acute stress study

Five Standardbred mares 9 to 14 years old belonging to the teaching herd at the Swedish University of Agricultural Sciences were included. The horses were not regularly transported, but all had previous experience of transportation and sample size was based on the number of horses that could be transported simultaneously on the truck. The experiments were conducted in the beginning of June (ambient temperature, 15°C‐25°C; humidity, 40%‐60%).

All horses underwent physical examination (including examination at walk) the day before the start of the study to ensure they displayed no signs of infectious or inflammatory disease. Thereafter, indwelling IV catheters were placed in the jugular vein.

On day 1, horses were sampled every hour between 8 am and 5 pm to obtain baseline results. After discarding 10 mL (>300% of combined catheter and extension set volume^17^), blood was collected from the IV catheter and placed in serum tubes. Catheters were thoroughly irrigated with heparinized saline after sampling. Blood samples were stored at room temperature for 90 to 120 minutes and then centrifuged at 5700*g* for 10 minutes. Serum was separated and stored at −80°C until analysis. Horses were housed in their regular stalls, and had regular turn‐out and feeding in individual pens during the day according to normal routine to minimize stress during baseline sampling.

On day 2, pre‐transport blood samples were collected at 8 am. Horses were loaded onto a truck and positioned perpendicular to the direction of transportation, tied up and separated by steel bars. The horses were transported for 1 hour between 9:20 and 10:25 am on a mixture of highway and countryside asphalt roads. After unloading, horses were placed in new, unfamiliar individual stalls with visual contact with each other and with free access to hay and water. Blood samples were collected as described above, directly after unloading and at 0.5, 1, 1.5, 2, 3, 4, 5, and 6 hours after arrival.

### 2.3 NGF ELISA

The NGF ELISA assay (Human beta‐NGF DuoSet ELISA DY256, R&D Systems, Minneapolis, Minnesota) was performed according to the kit manual. The pre‐determined detection range was 31.2 to 2000 pg/mL. Before analysis, recovery of equine NGF was tested by spiking equine serum with recombinant equine NGF (Nori Equine NGF‐B ELISA kit, Genorise Scientific, Philadelphia, Pennsylvania). Linearity experiments also were performed to determine optimal serum concentration for recovery. In addition to the standard curve, an aliquoted serum sample was run on all plates for normalization and comparison of results among plates. All samples were run in duplicate and mean values used for data analysis.

Briefly, 100 μL of sample (diluted 1:2) or standard was added to the plate, which had been coated with capture antibody. The plate was covered and incubated for 2 hours at room temperature. After washing, 100 μL of detection antibody was added to all wells and the plate was incubated for 2 hours at room temperature. After washing again, 100 μL streptavidin‐horseradish peroxidase (streptavidin‐HRP) was added and the plate was incubated for 20 minutes. After washing, color substrate solution was added and the plate incubated for another 20 minutes. Thereafter, 50 μL stop solution was added and the optical density was read within 5 minutes at 450 and 570 nm using a plate reader (Tecan Spark, Tecan Group Ltd., Männedorf). Magellan software (Tecan Group Ltd., Männedorf) was used for calculation of concentrations.

Recovery of equine recombinant NGF spiked in serum was 99.0% and the intra‐plate coefficient of variation (CV) ≤13% as calculated from all duplicate sample results. Data from the validation experiments is provided as Supporting Information (Data S1).

### 2.4 Cortisol analysis

Serum cortisol concentration was analyzed by immunoassay (Immulite 2000XPi, Siemens AB, Solna) and the analysis was performed by the Clinical Pathology Laboratory at the Swedish University of Agricultural Sciences. Mean results of sample duplicates were used for data analysis and CV was ≤9% as calculated from all duplicate sample results. Samples included for cortisol analysis were the samples obtained before transportation at 8 am, directly after unloading from the truck at 10:35 am and then at 0.5, 1.5, 3, 4, 5 and 6 hours after transportation. All of the corresponding day 1 baseline samples for comparison were obtained at the same time of day within a maximum time difference of 30 minutes between days.

### 2.5 Data analysis

Serum NGF concentrations for the lame and healthy cohorts were not normally distributed as analyzed using Shapiro‐Wilk testing and normal quantile plots, and therefore were compared using Kruskal‐Wallis and Mann‐Whitney *U* tests. Significance was set at *P* < .05. Correlation between age and serum NGF concentration was computed for the groups and tested using bivariate analysis. Influence of sex was tested using one‐way analysis of variance (ANOVA). Serum NGF concentrations in horses in the advanced osteoarthritis group with reaction to flexion tests in either 1 or all 4 limbs were compared using the Mann‐Whitney *U* test.

For analysis, values below the limit of detection were set at 31 pg/mL, just under the limit.

Receiver operating characteristic (ROC) analysis was performed. The area under the curve (AUC) was used to determine the specificity and sensitivity of the ELISA and the robustness of NGF in distinguishing between serum from sound horses and serum from lame horses with advanced osteoarthritis.

Statistical difference from baseline data for serum cortisol concentration was analyzed using the ANOVA mixed model for repeated sampling and least square means estimates with a 95% confidence interval (CI). Within‐day comparisons for serum NGF concentrations were analyzed using the same model, evaluating circadian rhythm for day 1 and the response to acute stress for day 2.

Statistical analyses were performed using the commercial software programs JMP Pro 14.0 (JMP Nordics, Marlow) and Graph Pad Prism 9.4 (Graph Pad Software, San Diego, California).

## 3 RESULTS

### 3.1 Lame cohorts

**FIGURE 1:**
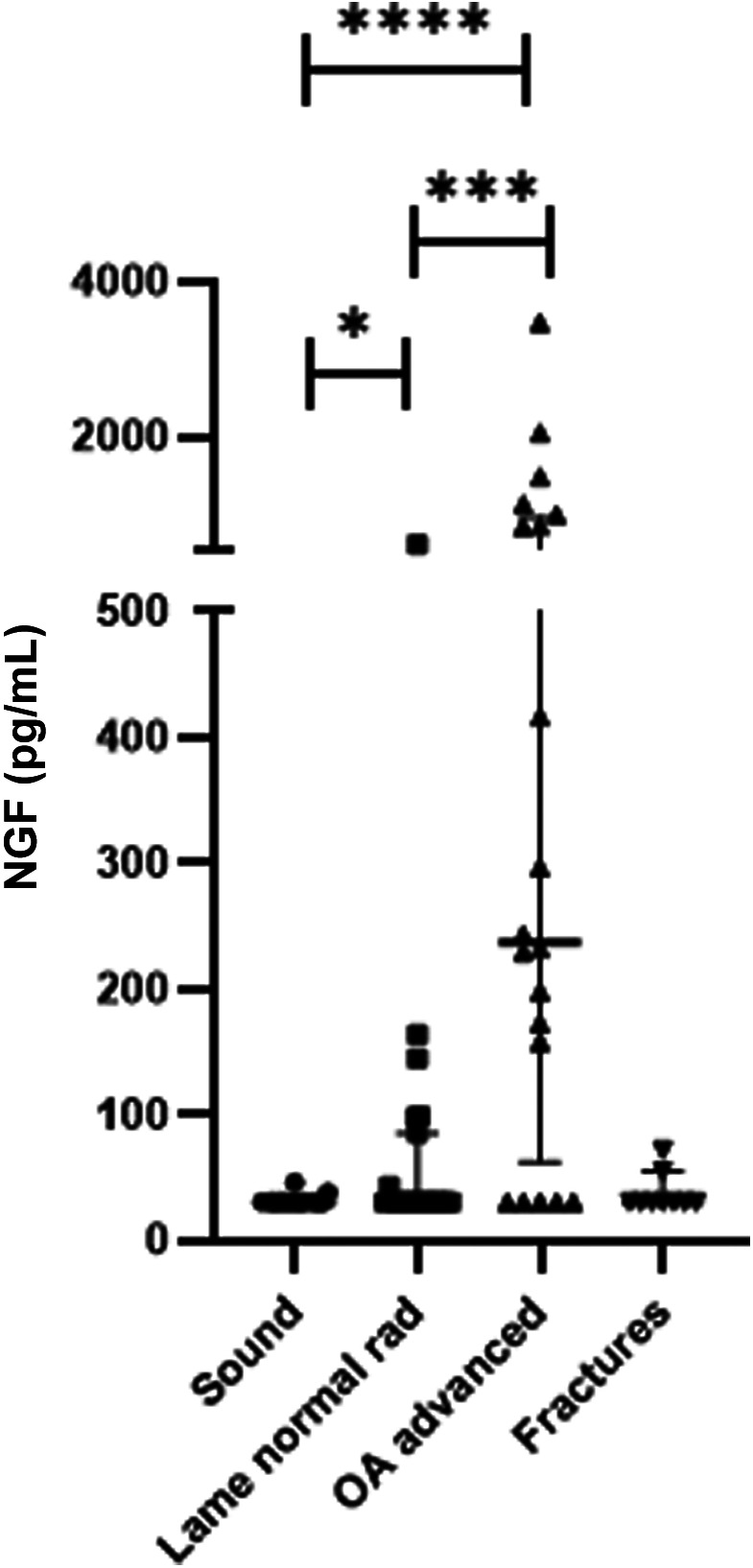
Serum NGF concentrations in lame horses with radiographic evidence of osteoarthritis (OA advanced, n = 20), lame horses without radiographic findings in the affected joint (Lame normal rad, n = 20), sound controls (Sound, n = 20), and painful horses with acute fractures (Fractures, n = 9). Bars mark median values with interquartile ranges. Both lame groups had higher NGF concentrations than the sound group. The group with advanced OA has significantly higher serum NGF than the group without radiographic findings. Horses with fractures are not included in the comparative analysis. *P < .05; ***P < .001; ****P < .0001.

**FIGURE 2:**
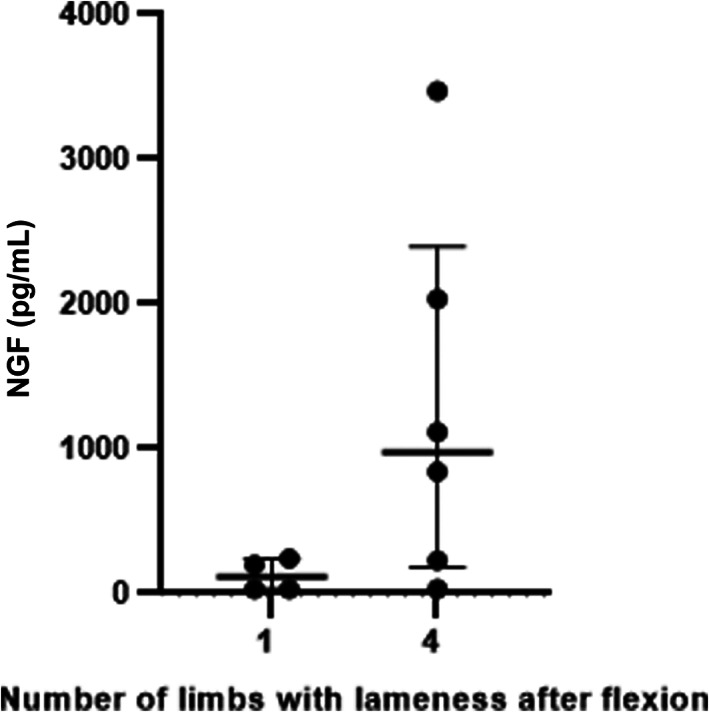
Serum NGF concentrations in horses with advanced osteoarthritis (radiographic changes) that had reaction to flexion in either 1 limb (n = 4) or 4 limbs (n = 6). Groups are not significantly different (*P* = .10). Bars mark medians with interquartile ranges.

Demographic data are presented in Table 1. In the advanced osteoarthritis group, 14 horses received intra‐articular anesthesia and 3 horses received regional anesthesia using nerve blocks. Three horses were not anesthetized. The joints determined to be the main cause of the lameness were carpal (n = 25), fetlock (n = 10), coffin (n = 3), and tarsal (n = 2). Serum NGF concentrations are presented as median and interquartile range (IQR). A significant difference was found in the serum NGF concentration between the advanced osteoarthritis group (median, 238 pg/mL; IQR, 63‐945 pg/mL) and the lame group without radiographic changes in the affected joint (median, 31 pg/mL; IQR, 31‐95 pg/mL). The sound group had significantly lower serum NGF concentrations (median, 31 pg/mL; IQR, 31‐46 pg/mL) than the lame groups (Figure 1). No correlation was found between age and serum NGF concentration within the lame groups, and concentrations were not influenced by sex (data not shown). Serum NGF concentrations in horses with advanced osteoarthritis with reaction to flexion in 1 vs all 4 limbs were not statistically different between groups (Figure 2).

**TABLE 1:** Demographic data for the four groups in the cross‐sectional study.

Diagnosis	Age (mean ± SD)	Sex (n)	Breed (n)
Advanced OA	12 ± 5.4	M (9), G (11)	Stb (1), Wb (12), Pony (5), PRE (1), Cross (1)
Lame, normal radiograph	4 ± 2.1	M (3), G (9), C (8)	Stb (20)
Healthy	1.4 ± 0.9	M (11), G (2), C (7)	Stb (17), Wb (3)
Fracture	6 ± 4.9	M (5), G (2), C (2)	Stb (4), Paint (1), Wb (1), Pony (3)

Abbreviations: C, Colt; G, Gelding; M, Mare; Paint, American paint horse; Pony, Various pony breeds; PRE, Pura Raza Española; Stb, Standardbred; Wb, Warmblood.

**FIGURE 3:**
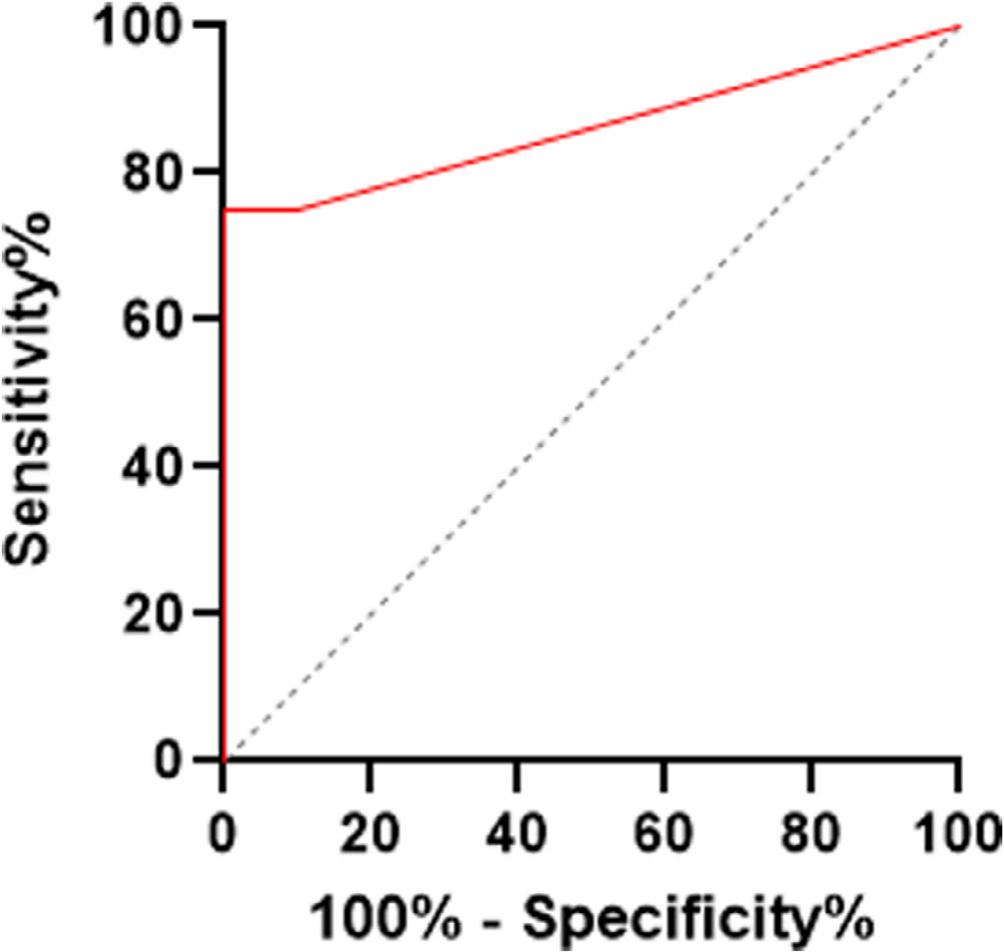
ROC curve of the specificity and sensitivity of the ELISA and the ability to separate lame horses with advanced osteoarthritis from sound horses by serum NGF concentrations. The area under the curve (AUC) is 0.86 (95% CI, 0.74‐0.99; *P* < .0001) indicating good separation of groups. The dotted line represents AUC = 0.5 which would be a result due to chance.

The ROC AUC was 0.86 (95% CI, 0.74‐0.99; Figure 3).

Nine horses with acute fractures were sampled; 5 were presented on the day of the traumatic event, 2 were presented the day after trauma, and 2 had a lameness history of 7 days. The horses with acute fractures were not included in the statistical comparisons because of smaller sample size, but serum NGF concentrations were low (median, 31 pg/mL; IQR, 31‐43 pg/mL).

### 3.2 Stress cohort

**FIGURE 4:**
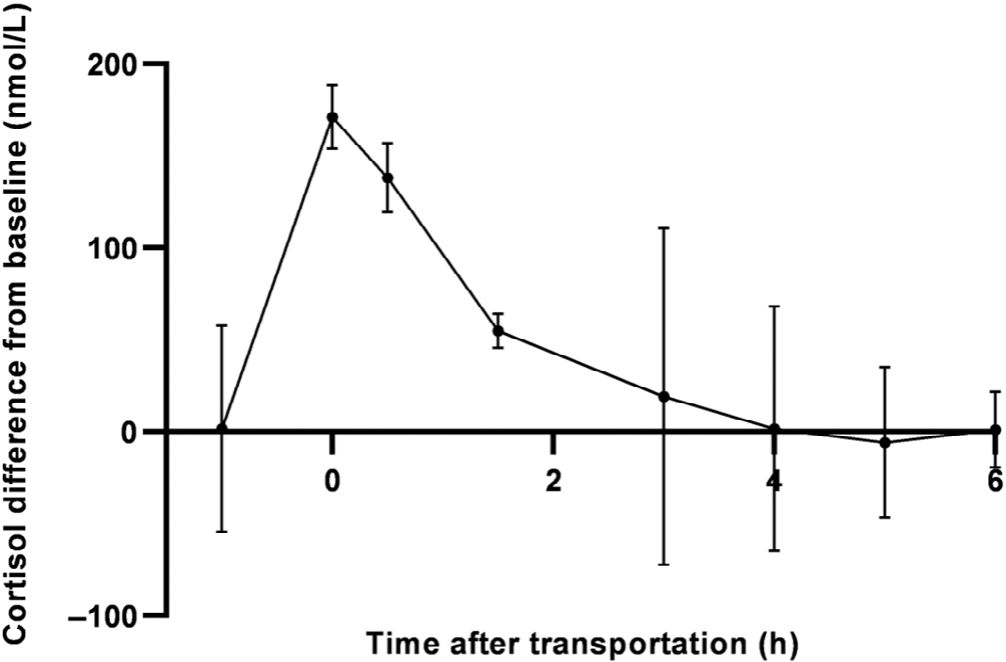
Difference in serum cortisol concentrations (mean ± SD) from baseline (cortisol concentration day 2 − cortisol concentration day 1) at each time point. Five horses were sampled 1 hour before loading, at unloading (*t* = 0) and at 6 time points after transportation, corresponding to the sampling times at day 1. Cortisol concentrations were significantly increased from baseline day 1 at 0, 0.5, and 1.5 hours after unloading. *P < .05.

Serum cortisol concentration was significantly increased from day 1 baseline at unloading (*t* = 0) and at 0.5 and 1.5 hours after unloading, confirming that transportation induced activation of the hypothalamic‐pituitary‐adrenal (HPA) axis consistent with a stress response (Figure 4).

For NGF, 2 horses had results above the upper limit of detection. These samples were re‐analyzed using 1:5 sample dilution to obtain readable results. One horse had serum NGF concentrations below the lower limit of detection throughout the study and therefore was excluded from statistical analysis. Short‐term stress induced by transportation did not cause changes in serum NGF concentrations (Data S2) and no circadian changes were detected during the day.

Detailed information on 95% CI for serum NGF concentrations at the different time points can be found in the Supporting Information (Data S3).

## 4 DISCUSSION

Nerve growth factor is associated with osteoarthritis and pain in humans,^8^, ^18^ but this association has not been well evaluated in horses. To the best of our knowledge, ours is the first study evaluating serum NGF concentrations in horses with osteoarthritis‐associated lameness. Our results show that, on a group level, serum NGF concentration is increased in horses with lameness associated with advanced osteoarthritis compared with sound horses and horses with milder disease. This finding is in agreement with a previous study in humans, where NGF concentration was found to be increased in serum of osteoarthritis patients undergoing knee replacement surgery compared with controls with minor meniscal injury.^8^ Nerve growth factor concentration has been shown to be increased in synovial fluid and chondrocytes from joints of horses with naturally‐occurring osteoarthritis.^19^ In a rat model of osteoarthritis, NGF increased in synovial membranes, chondrocytes, and subchondral bone after medial meniscectomy.^20^ Nerve growth factor can cause neuronal sensitization,^21^ it increases nerve growth and sprouting,^22^ and NGF transcription has been found to be increased in the dorsal horn of rats 8 weeks after destabilization of the medial meniscus.^23^ These findings indicate that NGF plays an active role in osteoarthritis‐associated pain both via local processes within the joint and in centralized pain adaptions in the dorsal horn of the spinal cord. Nerve growth factor induces joint inflammation and hypersensitization in experimental osteoarthritis.^24^, ^25^ However, monoclonal NGF antibody treatment does not stop disease progression. In fact, because of severe adverse effects with rapidly progressing osteoarthritis in some patients, the drug has not yet been approved for use in humans despite many years of clinical trials. The reason for the adverse reaction is still unknown. Concurrent nonsteroidal antiinflammatory drug treatment with impaired healing of joint tissue or overuse because of increased comfort have been suggested as underlying mechanisms.^26^ Whatever the reason, these adverse events indicate that serum NGF concentration increases as a result of osteoarthritis and is not a causative factor.

We do not know if the horses with advanced osteoarthritis in our study had centralized pain because no gold standard is available for measuring it in horses. Using lameness as a proxy for pain, horses displayed 1 sign of osteoarthritis‐associated pain. Extrapolating information from humans, where osteoarthritis can cause centralized pain and systemic pain states,^27^ we specifically selected the horses with advanced osteoarthritis that had reaction to flexion test in all 4 limbs and compared serum NGF concentrations in these individuals to those with advanced osteoarthritis that only showed reaction to flexion in 1 limb. This approach was used as an attempt to separate horses with a more generalized pain state from those that only displayed localized signs of pain. The difference between groups was not significant, which could have been a result of the small sample size because the analysis was underpowered (Figure 2). Horse owners were interviewed regarding disease duration, but this information was not reliable.^28^ Some of the owners were either completely unaware of the lameness or reported a lameness history of only a few days despite the fact that horses were presented with intra‐articular osteophytes, indicating that the disease likely had been unnoticed for several months.^29^ Therefore, no attempts were made to draw conclusions about disease duration and the finding of radiographic changes vs no changes was used as a proxy for duration. However, in humans with osteoarthritis, the severity of radiographic changes shows poor correlation with pain scores.^27^ Five horses had advanced osteoarthritis and did not have detectable serum NGF concentrations. Three of these horses had not received intra‐articular anesthesia, and it is possible that the clinical lameness in these individuals did not arise primarily from the osteoarthritic changes in the joint. All horses in the advanced osteoarthritis group had radiographic evidence of osteophytes, and thus serum NGF concentration is not solely dependent on the absence or presence of advanced osteoarthritic disease. It remains to be determined if serum NGF concentration is increased in certain osteoarthritis disease stages or pain states. Based on our study, on a group level, horses with advanced osteoarthritis have increased serum NGF concentrations. These increases do not appear to be caused by short‐term stress or acute pain.

Nerve growth factor can be secreted as a mature form (mNGF), or as a biologically active precursor (proNGF).^30^ The resultant effects of NGF vary not only with the balance of mNGF/proNGF, but also depend on interaction with 2 different receptors (tyrosine kinase receptor A [TrkA] and p75^NTR^). Binding to the TrkA receptor promotes nerve growth and survival. Binding to TrkA is enhanced by p75^NTR^, but binding to the p75^NTR^ receptor in conjunction with sortillin instead of TrkA can induce death and apoptosis. Studies have shown that mNGF has high affinity for TrkA receptors whereas proNGF preferentially binds to p75^NTR^ ^31^ and proinflammatory effects of the proNGF/p75^NTR^ interaction have been shown.^30^ Both receptors have impact on transmission of pain signals.^32^ It is not known what forms of NGF were detected with the ELISA used in our study,^33^ and it cannot be excluded that horses with acute pain (fracture group) expressed undetected proforms of NGF. Antibody information could not be obtained from the manufacturer because it was proprietary information. Attempts were made to determine the ELISA‐antibody affinity in serum by Western blotting and by capillary simple Western, but have so far been unsuccessful (data not shown).

Transportation did not influence serum NGF concentrations and horses with acute pain from fractures did not have high serum NGF concentrations. Although circadian rhythm was not specifically evaluated in our study, no daytime fluctuations were observed in the stress cohort baseline samples (Data S3). These findings suggest that serum NGF concentrations are not influenced by external factors, such as time of day or acute physiological stress, before sampling. It is not known how production and release of NGF are controlled. Previous studies in humans have shown that serum NGF concentration increases with emotional stress,^14^ and studies in mice showed increased serum NGF concentrations after intraspecific fighting,^15^ whereas studies in dogs have had contradictory results.^34^, ^35^ Previous studies on horses have led to the conclusion that serum NGF concentration increases with stress in this species, but these studies did not quantify NGF. These conclusions are based on the induction of nerve sprouting from either fetal chick ganglia or PC12 cells seen in vitro after incubation with equine serum, indicating activity of neurotrophic factors. In 1 study, nerve sprouting increased when cells were treated with post‐exercise serum,^36^ but this result could have been caused by other neurotrophins such as brain‐derived neurotrophic factor (BDNF).^37^ In another study, NGF activity in serum was increased in horses after transportation.^38^ NGF was confirmed as the cause of nerve sprouting by the addition of neutralizing NGF antibodies. However, nerve sprouting only occurred with serum from horses that were showing signs of infection after transportation and hence the increased NGF activity could have been associated with the inflammatory state of the horse.^5^

Our study had some limitations. The horses in the stress cohort were all sound at walk, but no full lameness examinations were performed before study inclusion. The horses were all retired race horses used for teaching, and some of them consistently showed signs of lameness when examined at a trot. It is likely that they had some extent of osteoarthritis at the time of examination, which could explain the high baseline results. Because the stress study was a longitudinal study where comparisons were only performed within each individual, the fact that possible osteoarthritis in this group was not determined is unlikely to have influenced our conclusions.

## 5 CONCLUSION AND CLINICAL IMPORTANCE

Marked differences were found between the group of sound horses and the group of lame horses with advanced osteoarthritis, with almost no detectable serum NGF in any of the sound horses. Furthermore, serum NGF concentration was not influenced by time of sampling or short‐term stress. Based on these findings, serum NGF concentration should be investigated as a marker for osteoarthritis‐associated pain.

Longitudinal studies of horses from the detection of early osteoarthritis without structural changes until more chronic, structural lesions develop would be helpful for determining changes in serum NGF concentration with disease progression. Additional studies also should be performed to evaluate the impact of systemic inflammation on serum NGF concentration. Previous findings of increased synovial fluid NGF in osteoarthritic equine joints^19^ in addition to our results indicate that monoclonal NGF‐antibody treatment could be an interesting alternative for future trials to find alternative treatments for osteoarthritis‐associated pain. Efforts should be made to determine if several forms of NGF are detectable in equine serum, because different forms of NGF could have different roles in acute and chronic pain and proNGF/mNGF ratios may vary with different diseases or disease stages.

## CONFLICT OF INTEREST DECLARATION

Authors declare no conflict of interest.

## OFF‐LABEL ANTIMICROBIAL DECLARATION

Authors declare no off‐label use of antimicrobials.

## INSTITUTIONAL ANIMAL CARE AND USE COMMITTEE (IACUC) OR OTHER APPROVAL DECLARATION

Sample collection was approved by the Ethical Committee on Animal Experiments, Uppsala, Sweden (Dnr: C154/4, Dnr: 5.8.18‐02896/2018 and Dnr: 5.8.18‐15533/2018). Written owner informed consent was obtained before sampling.

## HUMAN ETHICS APPROVAL DECLARATION

Authors declare human ethics approval was not needed for this study.

## Supporting information


**Data S1.** Supporting Information.

**Data S2.** Supporting Information.

**Data S3.** Supporting Information.
