# Supplementary material for: Serum nerve growth factor in horses with osteoarthritis‐associated lameness
Source: J Vet Intern Med. 2023 Apr 21;37(3):1201–8. doi: 10.1111/jvim.16718 (PMC10229367; doi:10.1111/jvim.16718)

Supporting Information S1

Data from the validation of the Human beta-NGF DuoSet ELISA DY256, R&D Systems, for the use in equine serum.

Prior to the experiments, alignment was performed (using the Uniprot online alignment system, https://www.uniprot.org/help/sequence-alignments) to compare the human NGF sequence to the equine NGF sequence. There was 94% homology between sequences and this was considered to provide a good chance for using antibodies directed at the human NGF protein.

1. First analysis of recombinant equine NGF (Standard from the Nori Equine NGF-B ELISA kit, Genorise Scientific) including information on the standard curve.

Sample	Concentration	% Recovery
Recombinant Equine NGF (100 pg)	130.66	130.66

Standard curve

Std expected conc	Concentration	% Recovery std curve
31.25	30.27	96.86
62.5	74.03	118.45
125	140.58	112.46
250	261.35	104.54
500	480.58	96.12
1000	958.02	95.80
2000	>max	>100

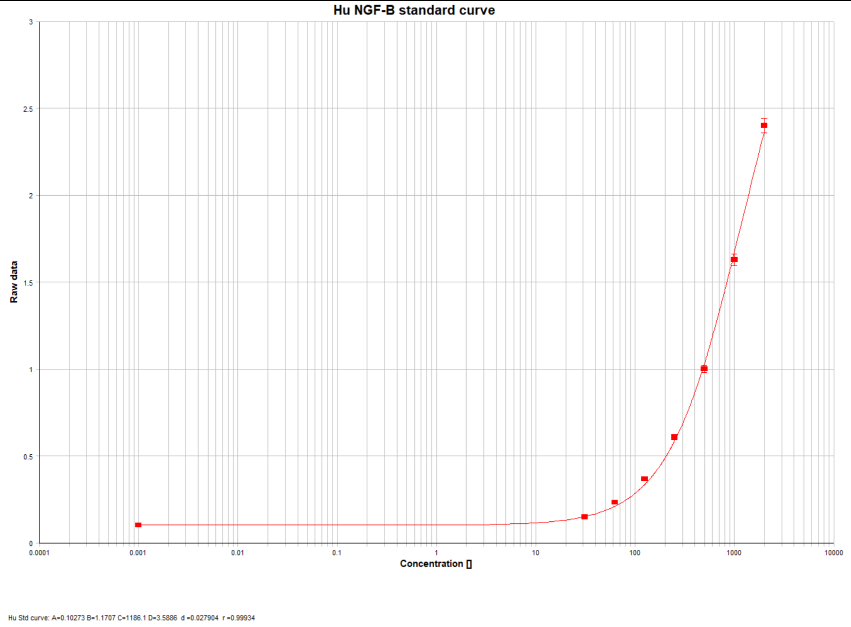

2. Dilution experiments, analysis of recombinant equine NGF (By using the standard from the Nori Equine NGF-B ELISA kit, Genorise Scientific) and analysis of equine serum spiked with 50 pg recombinant equine NGF. These experiments were performed at a) the conditions recommended by the manufacturer (room temperature, no shaking), b) capture as recommended, detection at 37° C with shaker, c) capture and detection at 37° C with shaker and d) capture at 37° C with shaker, detection as recommended. Based on these experiments it was decided to run the plates in the conditions recommended by the manufacturer with a serum dilution of 1:2.

OC: Operation control serum. This serum was later included at a 1:2 dilution in all plates used in the study.
% Recovery spiked serum = (observed conc of spiked sample – observed conc unspiked sample)/50 * 100
% Recovery Equine recombinant NGF = observed conc of sample/100 * 100

a) Recommended conditions

Sample	Concentration	% Recovery
Equine OC serum undiluted	53.14	
Equine OC serum 1:2	1327.09	
Equine OC serum 1:4	1120.24	
Equine OC serum 1:8	1745.23	
Recombinant equine NGF (100 pg)	130.34	130.34
Equine serum 1:2 dilution	382.21	
Equine serum 1.2 dilution spiked (50 pg recombinant equine NGF)	431.71	99.00

Standard curve

Std expected conc	Concentration	% Recovery std curve
31.25	31.91	102.1
62.5	65.44	104.7
125	130.74	104.6
250	250.04	100.0
500	500.19	100.0
1000	970.23	97.0
2000	>max	>100

b) Detection at 37° C with shaker

Sample	Concentration	% Recovery
Equine OC serum undiluted	59.97	
Equine OC serum 1:2	977.13	
Equine OC serum 1:4	622.04	
Equine OC serum 1:8	660.21	
Recombinant equine NGF (100 pg)	64.73	64.73
Equine serum 1:2 dilution	330.14	
Equine serum 1.2 dilution spiked (50 pg recombinant equine NGF)	364.66	69.04

Standard curve

Std expected conc	Concentration	% Recovery std curve
31.25	39.70	127.04
62.5	73.57	117.71
125	143.29	114.63
250	259.29	103.71
500	484.83	96.97
1000	935.14	93.51
2000	>max	>100

c) Capture and detection at 37° C with shaker

Sample	Concentration	% Recovery
Equine OC serum undiluted	12.63	
Equine OC serum 1:2	576.40	
Equine OC serum 1:4	647.88	
Equine OC serum 1:8	814.12	
Recombinant equine NGF (100 pg)	60.71	60.71
Equine serum 1:2 dilution	258.54	
Equine serum 1.2 dilution spiked (50 pg recombinant equine NGF)	296.87	76.66

Standard curve

Std expected conc	Concentration	% Recovery std curve
31.25	38.44	123.0
62.5	72.70	116.3
125	130.91	104.7
250	254.21	101.7
500	487.60	97.5
1000	949.63	95.0
2000	>max	>100

d) Capture at 37° C with shaker

Sample	Concentration	% Recovery
Equine OC serum undiluted	19.78	
Equine OC serum 1:2	630.00	
Equine OC serum 1:4	1017.80	
Equine OC serum 1:8	1783.06	
Recombinant equine NGF (100 pg)	118.16	118.16
Equine serum 1:2 dilution	356.83	
Equine serum 1.2 dilution spiked (50 pg recombinant equine NGF)	392.50	71.34

Standard Curve

Std expected conc	Concentration	% Recovery std curve
31.25	32.94	105.41
62.5	67.87	108.59
125	130.80	104.64
250	253.55	101.42
500	491.38	98.27
1000	974.09	97.41
2000	>max	>100

Standard curves from experiment a-d

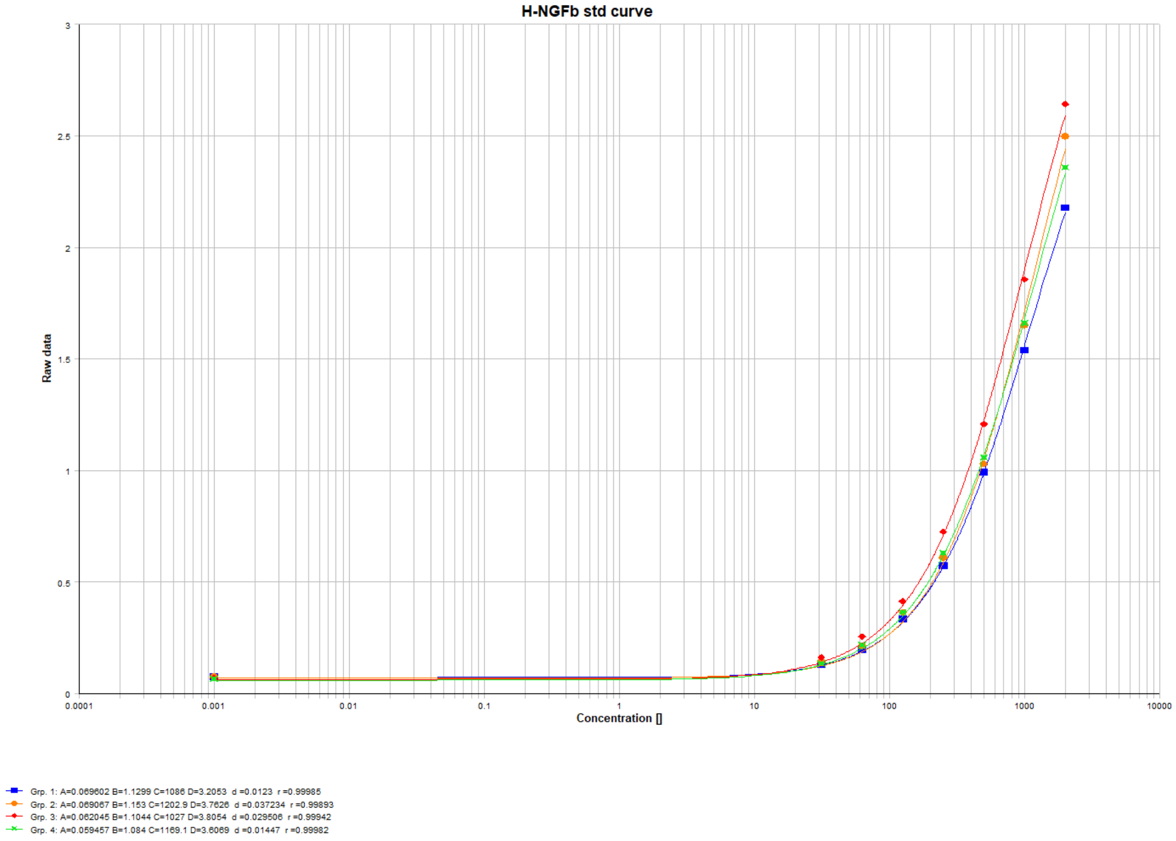

Supplement: Supplementary file 1 — Data S1. Supporting Information. [file JVIM-37-1201-s002.pdf]
