# Supplementary material for: Serum nerve growth factor in horses with osteoarthritis‐associated lameness
Source: J Vet Intern Med. 2023 Apr 21;37(3):1201–8. doi: 10.1111/jvim.16718 (PMC10229367; doi:10.1111/jvim.16718)

Supporting Information S2

Serum cortisol difference from baseline values (cortisol day 2 – cortisol day 1) and serum NGF concentrations displayed in individual diagrams for each horse. Sampling times were 1 hour prior to loading, at unloading (0), 1, 1.5, 3, 4, 5 and 6 hours after unloading. The y-axis is split so that cortisol (nmol/L) is plotted on the lower halft and NGF (pg/mL) is plotted on the upper half. Acute stress (confirmed by increased cortisol) did not increase serum NGF levels. There was a marked difference in basal NGF levels between horses and one horse in the cohort did not have measurable concentrations (data set not shown).

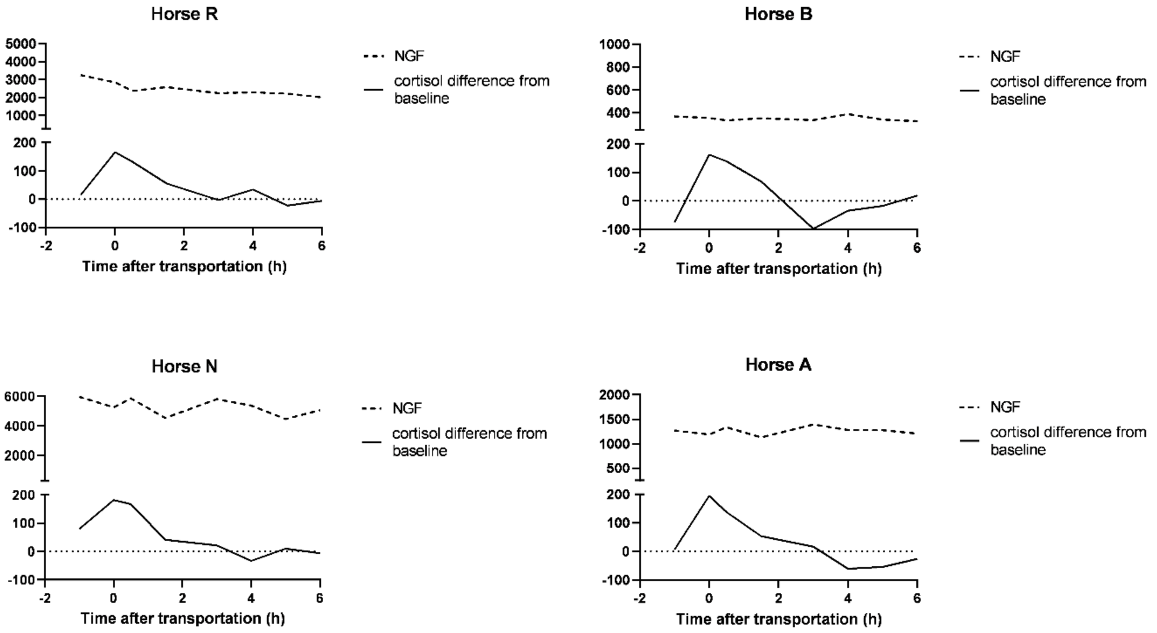

Supplement: Supplementary file 2 — Data S2. Supporting Information. [file JVIM-37-1201-s003.pdf]
