# Supplementary material for: Serum nerve growth factor in horses with osteoarthritis‐associated lameness
Source: J Vet Intern Med. 2023 Apr 21;37(3):1201–8. doi: 10.1111/jvim.16718 (PMC10229367; doi:10.1111/jvim.16718)

Supplementary Material Data S3

Results from the statitstical analysis of NGF data from the stress cohort (n=4)

Fixed Effects Parameter Estimates (Day 1, baseline)

Term	Estimate	Std Error	DFDen	t Ratio	Prob>|t|	95% Lower	95% Upper
Intercept	2806,1034	1318,1385	3,2	2,13	0,1187	-1275,25	6887,4565
Time (h) fixed[10-8]	321,32521	313,51831	20,6	1,02	0,3173	-331,4913	974,1417
Time (h) fixed[11-10]	-107,582	305,78213	16,0	-0,35	0,7295	-755,6539	540,48992
Time (h) fixed[12-11]	88,315462	305,78213	16,0	0,29	0,7764	-559,7564	736,38736
Time (h) fixed[13-12]	-600,0664	305,78213	16,0	-1,96	0,0673	-1248,138	48,005463
Time (h) fixed[14-13]	-108,0435	305,78213	16,0	-0,35	0,7284	-756,1154	540,0284
Time (h) fixed[15-14]	221,89534	305,78213	16,0	0,73	0,4785	-426,1766	869,96724
Time (h) fixed[16-15]	215,8792	305,78213	16,0	0,71	0,4903	-432,1927	863,95111

Fixed Effects Tests (Day 1, baseline)

Source	Nparm	DFNum	DFDen	F Ratio	Prob > F
Time (h) fixed	7	7	16,2	1,4161647	0,2651

Multiple Comparisons for Time (h) fixed (Day 1)
Least Squares Means Estimates

Time (h) fixed	Estimate	Std Error	DF	Lower 95%	Upper 95%
8	2806,1034	1318,1385	3,154	-1275,250	6887,4565
10	3127,4287	1318,1385	3,154	-953,924	7208,7817
11	3019,8467	1318,1385	3,154	-1061,506	7101,1997
12	3108,1621	1318,1385	3,154	-973,191	7189,5152
13	2508,0957	1318,1385	3,154	-1573,257	6589,4487
14	2400,0522	1318,1385	3,154	-1681,301	6481,4052
15	2621,9475	1318,1385	3,154	-1459,406	6703,3006
16	2837,8267	1318,1385	3,154	-1243,526	6919,1798

Fixed Effects Parameter Estimates (Day 2)

Term	Estimate	Std Error	DFDen	t Ratio	Prob>|t|	95% Lower	95% Upper
Intercept	2711,0597	1097,6123	3,1	2,47	0,0878	-728,8647	6150,9842
Time (h) fixed[10,5-8]	-297,5381	213,28919	26,8	-1,39	0,1745	-735,3549	140,27874
Time (h) fixed[11-10,5]	61,619203	213,04432	26,8	0,29	0,7746	-375,6855	498,92394
Time (h) fixed[11,5-11]	-72,57493	213,04432	26,8	-0,34	0,7360	-509,8797	364,72981
Time (h) fixed[12-11,5]	-254,3364	213,04432	26,8	-1,19	0,2430	-691,6416	182,96878
Time (h) fixed[12,5-12]	-21,3613	213,04432	26,8	-0,10	0,9209	-458,6785	415,95587
Time (h) fixed[13,5-12,5]	316,10298	213,28863	12,9	1,48	0,1624	-145,201	777,40695
Time (h) fixed[14,5-13,5]	-109,1813	213,28863	12,9	-0,51	0,6174	-570,4853	352,12267
Time (h) fixed[15,5-14,5]	-260,7497	213,28863	12,9	-1,22	0,2434	-722,0537	200,55439
Time (h) fixed[16,5-15,5]	78,067041	213,28863	12,9	0,37	0,7203	-383,2372	539,3713

Fixed Effects Tests (Day 2)

Source	Nparm	DFNum	DFDen	F Ratio	Prob > F
Time (h) fixed	9	9	16,4	1,6299484	0,1867

Multiple Comparisons for Time (h) fixed day 2
Least Squares Means Estimates

Time (h) fixed	Estimate	Std Error	DF	Lower 95%	Upper 95%
8	2711,0597	1097,6123	3,0839	-728,865	6150,9842
10,5	2413,5217	1097,6123	3,0839	-1026,403	5853,4460
11	2475,1409	1097,6123	3,0839	-964,783	5915,0652
11,5	2402,5660	1097,6123	3,0839	-1037,358	5842,4902
12	2148,2295	1097,6123	3,0839	-1291,695	5588,1540
12,5	2126,8682	1097,6123	3,0839	-1313,056	5566,7927
13,5	2442,9712	1097,6123	3,0839	-996,953	5882,8955
14,5	2333,7899	1097,6123	3,0839	-1106,134	5773,7142
15,5	2073,0402	1097,6123	3,0839	-1366,884	5512,9645
16,5	2151,1072	1097,6123	3,0839	-1288,817	5591,0316

Least Squares Means Plot (Day 1)

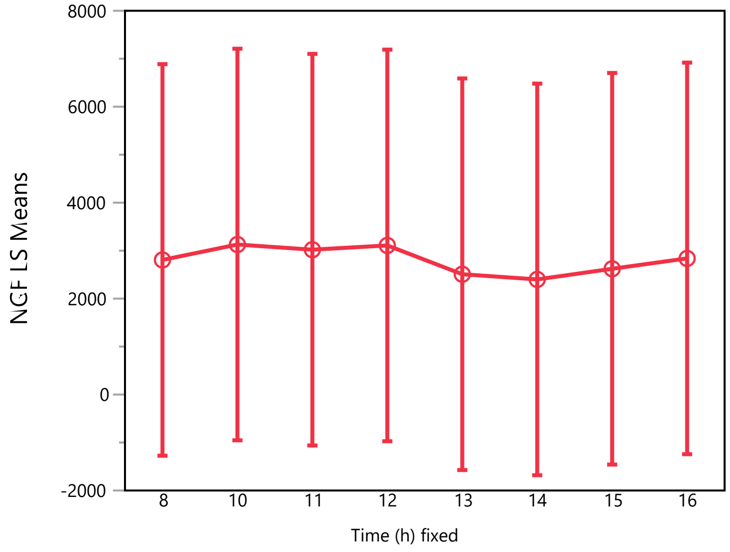

Least Squares Means Plot (Day 2)

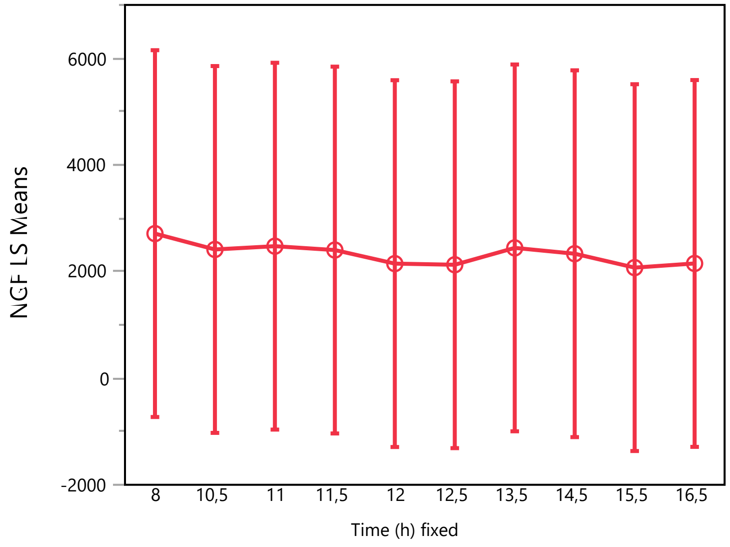

Supplement: Supplementary file 3 — Data S3. Supporting Information. [file JVIM-37-1201-s001.pdf]
